# Expression of the p53 Target Wig-1 Is Associated with HPV Status and Patient Survival in Cervical Carcinoma

**DOI:** 10.1371/journal.pone.0111125

**Published:** 2014-11-07

**Authors:** Li-Di Xu, Susanne Muller, Srinivasan R. Thoppe, Fredrik Hellborg, Lena Kanter, Mikael Lerner, Biying Zheng, Svetlana Bajalica Lagercrantz, Dan Grandér, Keng Ling Wallin, Klas G. Wiman, Catharina Larsson, Sonia Andersson

**Affiliations:** 1 Department of Oncology-Pathology, Cancer Center Karolinska (CCK), Karolinska University Hospital-Solna, Stockholm, Sweden; 2 Department of Women's and Children's Health, Division of Obstetrics and Gynecology, Karolinska University Hospital-Solna, Stockholm, Sweden; 3 Department of Molecular Medicine and Surgery, Karolinska Institutet, Karolinska University Hospital-Solna, Stockholm, Sweden; Rush University Medical Center, United States of America

## Abstract

The p53 target gene *WIG-1* (*ZMAT3*) is located in chromosomal region 3q26, that is frequently amplified in human tumors, including cervical cancer. We have examined the status of *WIG-1* and the encoded Wig-1 protein in cervical carcinoma cell lines and tumor tissue samples. Our analysis of eight cervical cancer lines (Ca Ski, ME-180, MS751, SiHa, SW756, C-4I, C-33A, and HT-3) by spectral karyotype, comparative genomic hybridization and Southern blotting revealed *WIG-1* is not the primary target for chromosome 3 gains. However, *WIG-1*/Wig-1 were readily expressed and *WIG-1* mRNA expression was higher in the two HPV-negative cervical cell lines (C33-A, HT-3) than in HPV-positive lines. We then assessed Wig-1 expression by immunohistochemistry in 38 cervical tumor samples. We found higher nuclear Wig-1 expression levels in HPV-negative compared to HPV positive cases (*p* = 0.002) and in adenocarcinomas as compared to squamous cell lesions (*p*<0.0001). Cases with moderate nuclear Wig-1 staining and positive cytoplasmic Wig-1 staining showed longer survival than patients with strong nuclear and negative cytoplasmic staining (*p* = 0.042). Nuclear Wig-1 expression levels were positively associated with age at diagnosis (*p* = 0.023) and histologic grade (*p* = 0.034). These results are consistent with a growth-promoting and/or anti-cell death function of nuclear Wig-1 and suggest that Wig-1 expression can serve as a prognostic marker in cervical carcinoma.

## Introduction

Cervical cancer ranks the third among the most common cancers in women worldwide and is the most common type of cancer in Eastern Africa, South-Central Asia and Melanesia [1]. The two major types are squamous cell carcinoma (SCC), accounting for around 80% of the cases, and adenocarcinoma (ADCA), for which the incidence has been increasing [2]–[4]. Persistent infection with high risk (HR) human papillomavirus (HPV) is a major risk factor for the development of cervical cancer and the two predominant types are HPV18 and HPV16, followed by HPV45, 31, 33, 58, 52, 35, 59, 56, 6, 51, 68, 39, 82, 73, 66 and 70 in order of prevalence [5].

HR-HPV encodes two early proteins, E6 and E7, that target two major cellular tumor suppressor pathways. E6 targets the p53 tumor suppressor for degradation, resulting in loss of p53-dependent apoptosis and/or senescence [6], [7]. E7 binds to the pRb tumor suppressor, thereby disrupting G1/S transition control [8]. Consequently, HR-HPV infection may lead to malignant transformation and tumor development.

Genomic alterations such as gene amplifications are important features of cervical carcinogenesis [9], [10]. Copy number gain in the long arm of chromosome 3 has been shown to be a biomarker of progression from carcinoma *in situ* to invasive cancer [11]. The amplified chromosome 3q region contains several genes with relevance to cancer, such as the phosphoinositide-3-kinase catalytic alpha polypeptide gene (*PIK3CA*) at 3q26.32 [12], the telomerase RNA component gene (*TERC*) at 3q26.2 [13], [14], the retinol-binding protein 1 and 2 genes (*RBP1-RBP2*) at 3q23, the *TP63* gene at 3q28 [15], and the sex-determining region Y-box 2 gene (*SOX2*) at 3q26.33 [16]–[18].

Human *WIG-1* (wild type p53-induced gene 1; also named *ZMAT3*) maps to 3q26.32 [19], which is in the critical 3q region. *WIG-1* is a bona fide p53 target gene [20]. It encodes a 288 amino acid nuclear zinc finger protein that binds to double-stranded RNA with high affinity [19], [21]. *WIG-1* is highly conserved from amoeba to human [22], [23] and the Wig-1 protein has been shown to bind to a U-rich element in the 3′-UTR of *TP53* mRNA which is then stabilized. Thus, Wig-1 forms a positive feedback loop with p53 that enhances p53 expression [24], [25]. Wig-1 can also bind to and stabilize the *MYCN* oncogene mRNA [26], and targets a number of other mRNAs [27]. Moreover, Wig-1 has been shown to destabilize *CDKN1A* mRNA (encoding p21) through recruitment of the RISC complex [28]. *WIG-1* is amplified and/or over-expressed in human papillary thyroid carcinoma (www.ebi.ac.uk/gxa/), lung squamous cell carcinoma [29], cervical SCC and other human tumors (www.cbioportal.org), suggesting an oncogenic function.

In this study we have examined the *WIG-1* gene in cervical carcinoma cell lines and Wig-1 expression in both cervical carcinoma cell lines and tumor samples. We investigated the expression of Wig-1 in relation to different clinical parameters such as: histological tumor type, grade and stage, HPV status, age at diagnosis, and survival. Our results indicate that high nuclear Wig-1 expression is a marker for poor prognosis in cervical carcinoma.

## Materials and Methods

### Established cell lines and analyses of chromosome 3 alterations

The 8 human cervical cancer cell lines Ca Ski, SiHa, C-4I, C-33A, ME-180, HT-3, MS751 and SW756 were purchased from ATCC (Table 1). The Saos2 cell line, originating from a human osteosarcoma was purchased from ATCC and used as a positive control for Wig-1 expression. Cells were cultured in DMEM or RPMI medium (GIBCO, Stockholm, Sweden) supplemented with 2 mM glutamine, 1% penicillin, and 1% streptomycin (GIBCO) with 10% newborn calf serum (GIBCO) at 37°C in the presence of 5% CO_2_.

**Table 1:** HPV status and chromosome 3 aberrations in the cell lines studied.

Cell line name	High risk HPV	Chromosome content		by SKY	by CGH***
*Cervical carcinoma cell lines*					
Ca Ski	HPV-16	(3n)	64–74	der(3)t(3;12)(p22;q24)	+3p12-qter(**q23–q26**)
ME-180	HPV-68*	(2n)	54–57	i(3)(q10)	+3q(**q27-qter**)
MS751	HPV positive**	(2n–3n)	37–75	der(3)t(3;8)(p11;q11)	-3pter-p11, +3q24-q26
SiHa	HPV-16	(3n)	65–71	der(X)t(X;?3)(p11;?)	+3p25-q26(**q23–q24**)
SW756	HPV-18	(2n)	37–45	none	+3q26
C-4 I	HPV-18	(2n)	35–45	none	+3q24–q26
C-33A	negative	(2n)	42–47	none	+3p21, +3q26
HT-3	negative	(3n)	57–59	der(3)t(3;12)(p11;q11)	-3p, +3q26–q27
*Osteosarcoma cell line*					
Saos2	negative	(2n–4n)	52-99	dup(3)(q12q29); dic(3;19)(?;q13.3); der(X)t(X;1;3)(X?->cen->X?::1?->1?::3?->3?)	+3q

* HPV-39 according to ATCC;

** HPV type was undetermined after DNA sequencing.

*** Bold in parenthesis indicates amplification within the gained interval.

Cell lines were HPV typed using a broad spectrum PCR assay [30], [31] and sequencing. Copy number alterations of chromosome 3 were analysed by comparative genomic hybridization (CGH) and spectral karyotyping (SKY) according to published methods [32], [33]. Gain of the *WIG-1* locus relative to a control probe from 3p23 was determined by Southern blot analysis using standard methods. The methods used are described in detail in the Supplementary material.

### *WIG-1* gene expression by Northern blot analysis and qRT-PCR

For Northern blot analyses cells were harvested by trypsination and total RNA was isolated using Trizol reagent (Invitrogen, Carlsbad, CA, USA) according to manufacturer's protocol. RNA was separated in 1% agarose/formamide gels and blotted to Zeta-Probe GT Genomic Tested Blotting membranes (Bio-Rad, Hercules, CA, USA). Probes used for hybridization were the coding part of *WIG-1* cDNA and a 500 bp PCR fragment of *beta-actin* labeled with the Megaprime DNA labeling System (GE Healthcare, Uppsala, Sweden). Hybridization was performed in ULTRA hybridization buffer (Life Technologies, Stockholm, Sweden) and the signals were detected and analyzed in a FLA-3000 phosphorimager (Fujifilm, Stockholm, Sweden). Normal human fibroblasts were used as control with *18S* RNA as a reference for the analysis.

*WIG-1* expression was also analysed by real-time PCR using *HPRT1* as reference gene and SYBR Green based LightCycler PCR as further described in the Supplementary material.

### Western blot analysis of cell lines

Cells were harvested by trypsination and lysed in a buffer containing 100 mM Tris pH 8.0, 150 mM NaCl, and 1% NP-40 and 1% Protease Inhibitor Cocktail (Sigma-Aldrich, Stockholm, Sweden). The protein lysates were separated in 10% SDS-PAGE gels and blotted at 20 V for 30 min to Hybond ECL-membranes (GE Healthcare, Stockholm, Sweden) using a transblot SD semi dry transfer cell (Bio-Rad, Hercules, CA). Primary antibodies used a human Wig-1 polyclonal antibody raised against a GST-Wig-1 fusion protein (1∶2000, [19]) and β-actin clone AC-15 (1∶5000, Sigma, Stockholm, Sweden). Signals were detected by using Super Signal West Femto maximum Sensitivity Substrate (Thermo Scientific, Stockholm, Sweden) with a CCD camera (Fujifilm, Stockholm, Sweden) and analysed by Luminescent Image Analyser LAS-1000 plus (Fujifilm, Stockholm, Sweden).

### Cervical tumor cases

We included formalin-fixed paraffin-embedded tissue specimens from 38 patients diagnosed and surgically treated at the Karolinska University Hospital during the period 1989–2010. The histopathological diagnosis was based on the WHO criteria. Thirteen cases had a squamous cell lesion classified as squamous cell carcinoma (SCC n = 9) or cancer *in situ* (CIS n = 4), and 25 cases had adenocarcinoma (ADCA). The 25 ADCAs were all without any squamous cell component. Furthermore, the HPV-negative tumors were histopathologically re-evaluated which revealed typical morphology in agreement with the initial diagnosis. Ethical approval was obtained from the Regional Ethical Review Board in Stockholm. Written informed consent was given by participants for their clinical records to be used in this study and patient records/information was anonymized and de-identified prior to analysis. All patients were retrospectively followed-up from the time of diagnosis until January 2012. Clinical information was obtained for each case concerning age at diagnosis, tumor classification, histology grade, stage and outcome after follow-up (Table S1). Serial sections were cut from each block and individually subjected HPV typing using established methods [34]–[36] as described in detail in the Supplementary material, as well immunohistochemistry.

### Validation of the Wig-1 antibody for immunohistochemistry

Total protein was extracted from a cervical carcinoma sample by homogenization with polytron system PT1200CL (Kinematica AG, Lucerne, Switzerland) and purified with AllPrep RNA/Protein Kit (Qiagen, Stockholm, Sweden). Protein concentrations were determined using Bradford (Bio-Rad, Stocholm, Sweden) and 30 to 50 µg were loaded on 10% NuPAGE Novex Bis-Tris Gels (Invitrogen, Stockholm, Sweden) in loading buffer and reducing agent (Invitrogen) and run in Mops buffer (Invitrogen). Proteins were blotted to nitrocellulose membranes using the iBlot Dry Blotting System (Invitrogen) and incubated with a rabbit polyclonal Wig-1 antibody (1∶2000, Genetex) followed by anti-β-actin antibody (1∶5000; Sigma–Aldrich). Primary antibodies were visualized using HPR-conjugated secondary antibodies (GE Healthcare, Stockholm, Sweden) and signals were detected as above.

For the purpose of immunohistochemistry the antibody was tested in a dilution serie from 1∶200 to 1∶1,600 which revealed that 1∶400 was the optimal dilution to assess Wig-1 expression. Furthermore different batches of the Wig-1 antibody from Gentex gave similar staining patterns in the cervical carcinoma samples.

### Immunohistochemistry

Immunohistochemistry was performed on all 38 cervical carcinoma samples. Sections were deparaffinized, rehydrated and microwaved in 10 mM citrate buffer (pH 6.0) for 20 minutes. Endogenous peroxidase activity was inhibited using 0.5% hydrogen peroxide (H_2_O_2_) in distilled water for 30 minutes. Non-specific staining was blocked by incubating the sections with 4% normal goat serum (DAKO, Stockholm, Sweden) in Tris buffer (TBS) for 30 minutes at room temperature. Sections were incubated with Wig-1 antibody (1∶400, Genetex, Irvine, USA) at 4°C overnight. On the following day the sections were washed in TBS and first incubated with goat anti-rabbit IgG (1∶200, DAKO, Stockholm, Sweden) and subsequently with ABComplex/HRP (DAKO, Stockholm, Sweden) in TBS with Triton (2∶2∶100) for 30 minutes at room temperature to visualize reaction products. Diaminobenzidine was used as chromogen. After counterstaining with hematoxylin, the slides were dehydrated and mounted with a xylene-soluble medium.

A senior pathologist, blinded for all clinical data, independently evaluated immunohistochemistry staining. Scoring of immunohistochemistry results was based on both nuclear and cytoplasmic staining intensity. Staining intensity was evaluated separately in nuclei and cytoplasm using a four-graded scale as no staining, weak, moderate, or strong staining.

### Statistical Analysis

Statistical analysis was performed using IBM SPSS 20.0. For statistical analysis, age data were categorized into three different ranges: <36 years, 36–55 years, and >55 years at diagnosis. Univariate analysis of variance was performed to assess association between either nuclear or cytoplasmic Wig-1 staining intensity with tumor types, HPV status and all clinical data including age at diagnosis, histologic grade and tumor stage. A p-value <0.05 was considered statistically significant. Survival was evaluated using the log-rank test and illustrated by Kaplan-Meier plots.

## Results

### The *WIG-1* locus is not a primary target for chromosome 3 genomic alterations in cervical carcinoma cell lines

**Figure 1:**
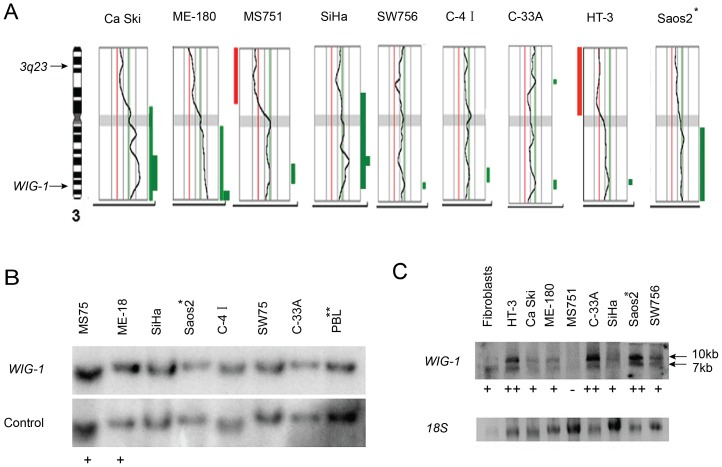
The *WIG-1* locus is not a primary target for genomic alternations of chromosomes 3 in cervical carcinomas. (A) CGH profiles of chromosome 3 in the eight cervical carcinoma cell lines and one control cell line (Saos2). Gains of chromosomal material are indicated by a bar to the right of the profile, and losses are marked to the left. A bold line indicates a region with amplification. The location of the probes for Southern analyses are shown next to the ideogram at the left. (B) Autoradiograms of Southern blot of cell line DNA after cleavage with EcoRI and hybridization with WIG-1 and subsequently with a central probe from 3p23. The names of the respective cell-lines are given above each lane. DNA from peripheral blood leukocytes (PBL) from a healthy donor was included as a normal reference. ‘+’ indicates relative gain of *WIG-1* as compared to PBL. (C) Northern blot demonstrating *WIG-1* mRNA levels in cervical carcinoma cell lines and the osteosarcoma cell line Saos2. Phosphorimage of Northern blot membrane hybridized with *WIG-1* and subsequently with *18S* as a loading control. Fibroblasts were included as reference. The two differently sized *WIG-1* transcripts are indicated to the right. The expression levels were classified as negative (-), moderate (+) and strong (++). *Saos2 and **PBL were used as control cell lines.

We first assessed structural and numerical alterations of the *WIG-1* locus in cervical carcinoma cell lines by SKY and CGH analyses, which showed that *WIG-1* is not a primary target for chromosome 3 alterations in these cells (Figure 1 A, Table 1). We found single structural chromosome 3 abnormalities by SKY in six of the cell lines including translocation derivatives, one iso-chromosome, and more complex rearrangements (Table 1). In no case did the breakpoint involve the 3q26.32 region where *WIG-1* is located. All nine lines studied by CGH exhibited copy number alterations of chromosome 3 (Figure 1A; Table 1). Amplifications were found in Ca Ski, ME-180 and SiHa that involved 3q23–26, 3q27-ter and 3q23–24, respectively. In addition, in two lines (MS751 and HT-3), loss of all or most of the short arm 3p was found. All cell lines studied exhibited complete or partial gain of 3q, with an overlapping region of gain located at 3q26.

We next studied the *WIG-1* locus for possible copy number alterations (Table 2). Using Southern analysis, *WIG-1* was compared with a control marker from the short arm of the same chromosome (“3p23”) (Figure 1B). This showed modest gain of *WIG-1* in MS751 and ME-180 cells, but no alterations in SiHa, SW756, C-4I, C-33A or Saos2.

**Table 2:** Copy number and expression of *WIG-1/*Wig-1 in relation to HPV infection and *TP53/p53* status.

		*WIG-1* copy number	Wig-1/*WIG-1* expression				
Cell line	HPV infection	by Southern	Wig-1	*WIG-1* by		Published *TP53/*p53 data **	
		*WIG-1* vs. *3p23*	by Western	qRT-PCR*	Northern	*TP53* sequence	p53 expression
*Cervical carcinoma cell lines*							
Ca Ski	Yes	n.d.	positive	3.07	+	wild-type	very low/absent
ME-180	Yes	gain	positive	4.91	+	wild-type	very low/absent
MS751	Yes	gain	positive	5.95	-	wild-type	absent
SiHa	Yes	no gain	positive	2.75	+	wild-type	very low/absent
SW756	Yes	no gain	positive	3.13	+	n.a.	n.a.
C-4 I	Yes	no gain	positive	4.67	n.d.	wild-type	very low/absent
C-33A	No	no gain	positive	5.79	++	mutated (p.Arg273Cys)	high but inactive
HT-3	No	n.d.	positive	5.47	++	mutated (p.Gly245Val)	high but inactive
*Osteosarcoma cell line*							
Saos2	No	no gain	positive	11.74	++	homozygously deleted	absent

-  =  negative;

+  =  detectable to moderate expression;

++  =  strong expression;

n.d.  =  not determined;

n.a.  =  not available;

* Given in arbitrary units as compared to fibroblasts (1.0).

** Reiss *et al.*, 1992; Yaginuma *et al.*, 1991; Srivastava *et al.*, 1992; Scheffner *et al.*, 1991; Masuda *et al.*, 1987.

Taken together, our analyses showed that 3q amplifications occur in cervical cancer cells, but *WIG-1* is not a primary target for frequent gains. This is well illustrated by the fact that *WIG-1* is located telomeric of the commonly amplified 3q23–24 interval in Me180 and SiHa cells, and centromeric of the amplified 3q27-ter interval in ME-180 cells. Similarly, these 3q amplifications detected by CGH were not accompanied by *WIG-1* locus amplifications as determined by Southern blotting.

### Elevated *WIG-1* expression in HPV-negative cervical carcinoma cell lines

We assessed *WIG-1* mRNA and Wig-1 protein expression in all cell lines (Table 2, Figure 1C, Figure S1 in File S1) by Northern and Western blotting. The two HPV-negative lines HT-3, C-33A and the control Saos-2 showed strong *WIG-1* mRNA expression, while the HPV-positive cell lines Ca Ski, ME-180, SiHa and SW756 showed weaker but clearly detectable expression. Two transcripts (10 kb and 7 kb) were identified in all cell lines, with predominance of the 10 kb mRNA (Table 2, Figure 1C). Similarly, *WIG-1* mRNA expression, as assessed by qRT-PCR, was higher in the two HPV-negative lines (C33-A, HT-3) than in the HPV-positive lines (Table 2, Figure S2 in File S1).

Western blotting showed Wig-1 protein expression in all cell lines (Table 2, Figure S1 in File S1). However, no associations were found between *WIG-1* expression levels and copy number alterations at the DNA level. We found no association between Wig-1 protein expression and HPV infection.

### Wig-1 protein expression in cervical carcinoma patient samples

**Figure 2:**
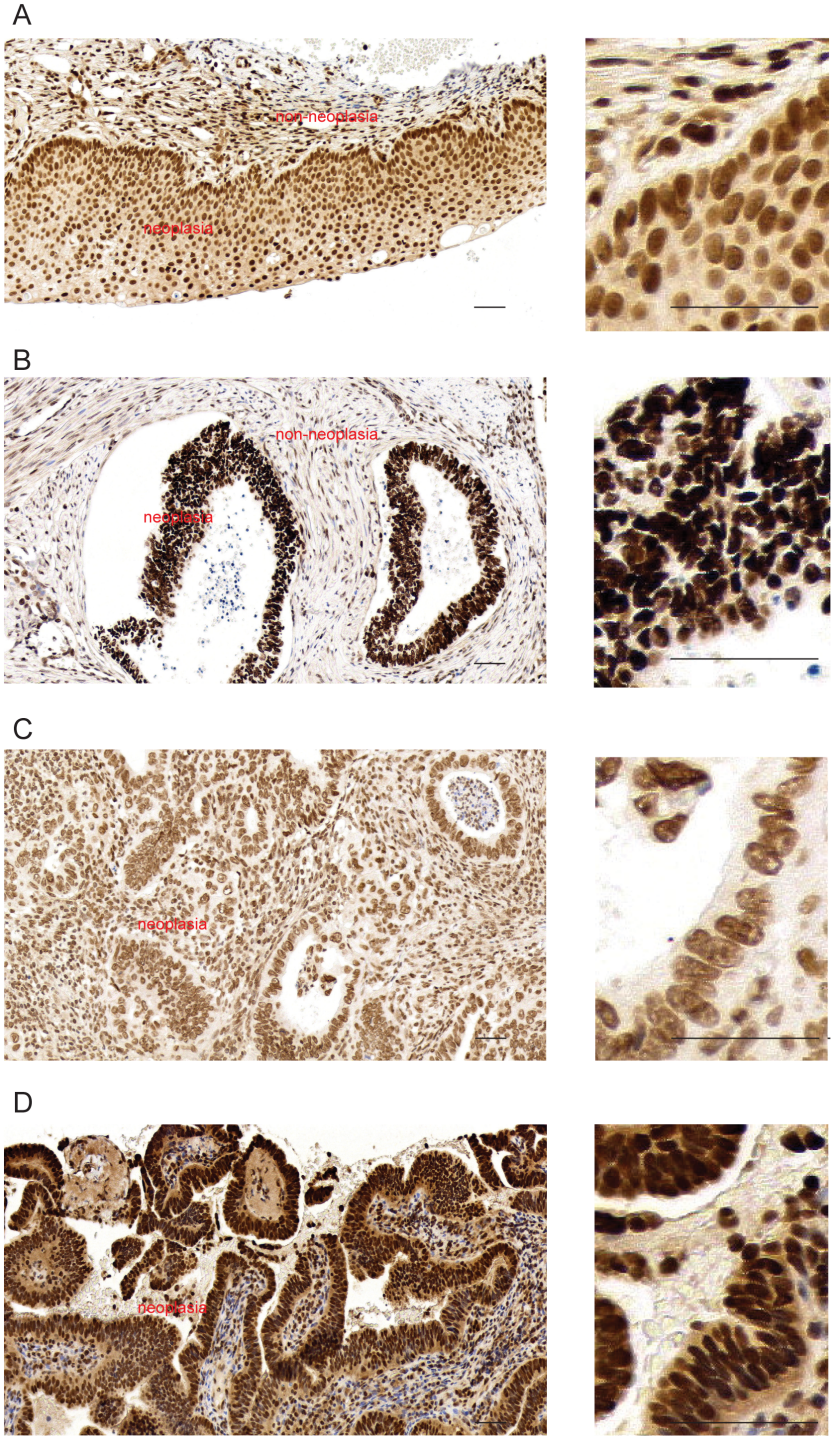
Wig-1 expression in cervical carcinomas. Immunohistochemical (IHC) analysis revealed four main Wig-1 staining patterns: (A) moderate nuclear and positive cytoplasmic Wig-1 staining (IHC staining picture of case CIS-3 as a representative) at low magnification (left) and high magnification (right); (B) strong nuclear and negative cytoplasmic Wig-1 staining (IHC staining picture of case ADCA-18 as a representative) at low magnification (left) and high magnification (right); (C) moderate nuclear and negative cytoplasmic Wig-1 staining (IHC staining picture of case ADCA-21 as a representative) at low magnification (left) and high magnification (right); and (D) strong nuclear and positive cytoplasmic Wig-1 staining (IHC staining picture of case ADCA-22 as a representative) at low magnification (left) and high magnification (right). Neoplasia and non-neoplasia are marked in all figures. Scale bar  = 50 µm.

We further examined Wig-1 protein expression in primary cervical carcinomas by immunohistochemistry. The specificity of this antibody was verified by demonstration of a single product of the expected size by Western blotting (Figure S3 in File S1). All 38 cases in this study showed positive Wig-1 staining, mainly in cell nuclei (Figure 2, Table S1). The Wig-1 staining intensities were evaluated in the nuclei and cytoplasm separately and classified as: no staining, weak, moderate and strong. Nuclear staining was observed in all 38 cases; 45% showed moderate and 55% showed strong staining (Table 3). Overall, the cytoplasmic Wig-1 staining intensity was relatively low compared to the nuclear staining; 58% of tumors were negative, whereas 42% were positive with weak cytoplasmic staining (Table 3) (From now on, we will use positive to refer to weak cytoplasmic Wig-1 staining and negative to refer to no cytoplasmic Wig-1 staining). We observed four different patterns of Wig-1 immunostaining: A) moderate nuclear and positive cytoplasmic staining; B) strong nuclear and negative cytoplasmic staining; C) moderate nuclear and negative cytoplasmic staining; and D) strong nuclear and positive cytoplasmic staining. Out of the 38 tumors, 8 showed pattern A, 13 showed pattern B, 9 showed pattern C, and 8 showed pattern D (Figure 2 A–D). Non-tumorous tissue adjacent to the tumors showed moderate nuclear Wig-1 staining and weak or no cytoplasmic Wig-1 staining.

**Table 3:** Comparison of Wig-1 IHC expression with clinical parameters.

		Nuclear Wig-1 staining (no. of cases)			Cytoplasmic Wig-1 staining (no.of cases)		
Parameters							
(informative n = )	No. of cases	moderate	high	P -value	no	weak	P -value
*Tumor type* (n = 38)				**<0.0001**			0.303
Squamous cell lesion	13	11	2		6	7	
Adenocarcinoma (ADCA)	25	6	19		16	9	
*HPV high risk* (ADCA, n = 25)				**0.049**			0.29
negative	13	1	12		7	6	
positive	12	5	7		9	3	
*HPV high risk* (all, n = 38)				**0.002**			0.92
negative	17	3	14		10	7	
positive	21	14	7		12	9	
*Age at diagnosis* (n = 38)				**0.023**			0.55
<36 years	5	5	0		2	3	
36–55 years	23	9	14		13	10	
>55 years	10	3	7		7	3	
*Histology grade* (n = 37)				**0.034**			0.407
CIN3/CIS	4	4	0		1	3	
- low	12	7	5		6	6	
- moderate	8	3	5		6	2	
- high	13	3	10		8	5	
*Stage* (n = 38)				0.071			0.058
CIS	4	4	0		1	3	
Stage 1	25	11	14		15	10	
Stage 2	3	1	2		2	1	
Stage 3	6	1	5		4	2	

CIN3  =  Cervical intraepithelial neoplasia 3; CIS  =  Cancer *in situ*.

Univariate analysis of variance were performed. P-values <0.05 were regarded as sgnificant and are indicated in bold.

In this panel of cervical carcinomas we observed a possible correlation between Wig-1 expression levels and HPV status. HPV DNA is known to almost invariably be present in SCC [37]. However, our patient tumor material included 4 HPV-negative squamous cell lesions (3 SCC and 1 CIS). When taking all squamous cell lesions into account, nuclear Wig-1 staining intensity was significantly different between the HPV-positive and HPV-negative cases (*p* = 0.002, univariate analysis). We also investigated whether the expression levels of Wig-1 differed between HPV-positive and HPV-negative adenocarcinomas (ADCA) and found a statistically significant difference in nuclear Wig-1 staining intensity between HPV-positive and HPV-negative ADCA samples (*p* = 0.049, univariate analysis). In general, HPV-negative cervical tumors showed higher nuclear Wig-1 expression levels than HPV-positive tumors.

### Association between nuclear Wig-1 expression and survival

Univariate analysis showed significant positive associations between nuclear Wig-1 staining intensity and clinical parameters, including age at diagnosis (*p* = 0.023) and histologic grade (*p* = 0.034) (Table 3). However, cytoplasmic Wig-1 staining intensity was not associated with any of those clinical parameters. In addition, nuclear Wig-1 staining intensity was significantly higher in ADCA as compared to squamous cell lesions (*p*<0.0001).

**Figure 3:**
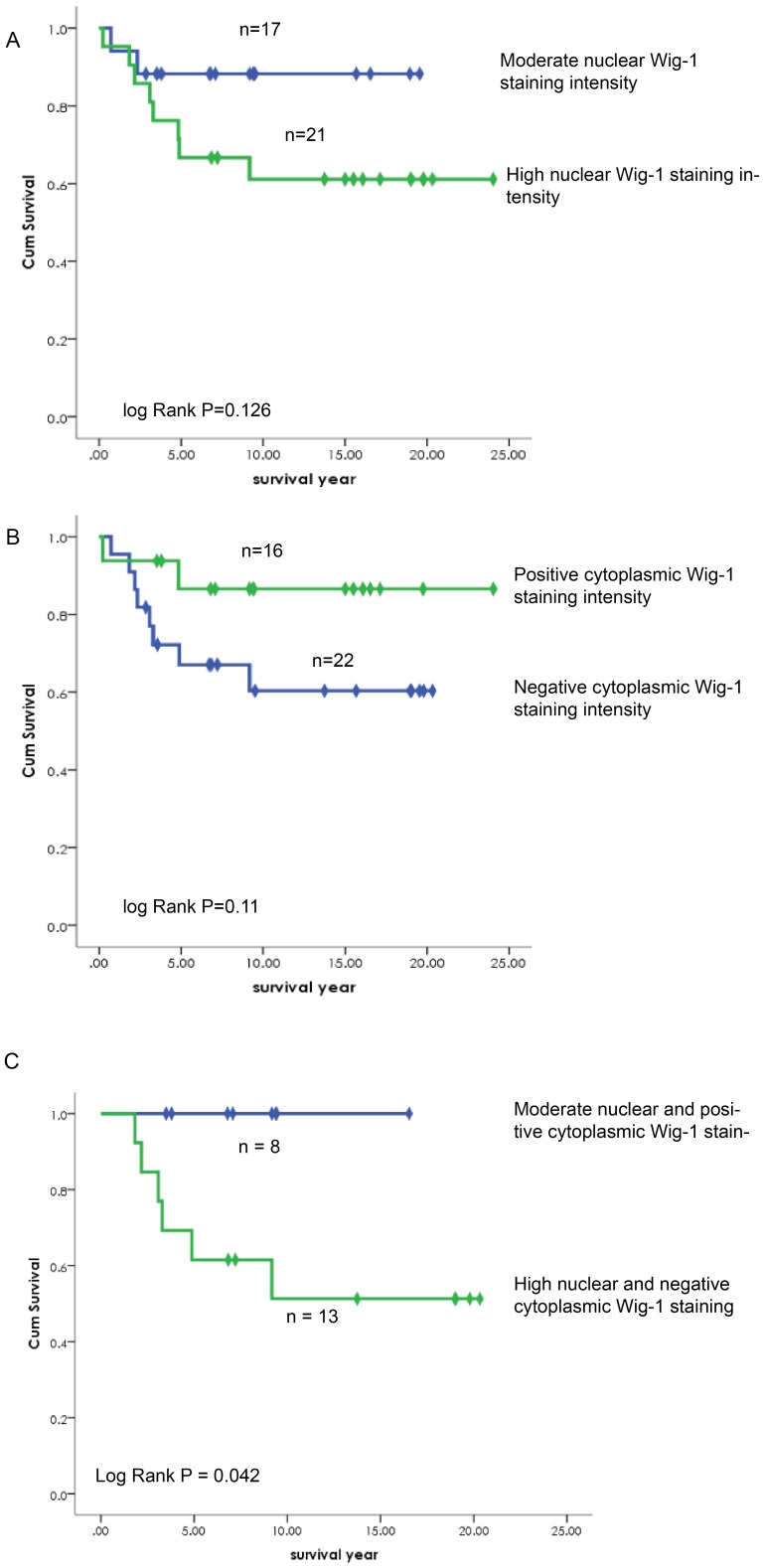
Kaplan-Meier survival curves showing cervical carcinoma patient survival as a function of Wig-1 staining pattern. (A) Patients with moderate nuclear Wig-1 staining show better survival than patients with high nuclear staining. (B) Patients with positive cytoplasmic Wig-1 staining have better survival than patients with negative cytoplasmic staining. (C) Patients with moderate nuclear and positive cytoplasmic Wig-1 staining show better survival than patients with strong nuclear and negative cytoplasmic staining. Cases no. and Log Rank P values are indicated. *p*<0.05 is considered to be significant.

Interestingly, we found that moderate nuclear Wig-1 expression was associated with better overall survival (Figure 3A). Positive cytoplasmic Wig-1 expression levels were also associated with better overall survival (Figure 3B).

We then examined survival in the subgroup of patients with tumors that demonstrated moderate nuclear and positive cytoplasmic Wig-1 staining (pattern A). We found that this subgroup of patients had a significantly better prognosis than patients whose tumors showed strong nuclear and negative cytoplasmic Wig-1 staining (pattern B; *p* = 0.042) (Figure 3C).

## Discussion

*WIG-1/*Wig-1 is amplified and/or overexpressed in many human tumors, including lung squamous cell carcinoma [29] and cervical squamous carcinoma (www.cbioportal.org), which is consistent with an oncogenic function. *WIG-1* has been mapped to chromosome 3q26.32, a region that shows frequent copy number gain in various human tumors [19]. Chromosome 3q gain occurs during progression from cervical carcinoma in situ to severe invasive cancer [11]. The value of detection of genomic amplification of 3q as a biomarker for progression during uterine cervix tumorigenesis was evaluated in several studies. Heselmeyer-Haddad et al. established the central role of 3q for progression from low-grade dysplastic lesions to higher grades and to invasive carcinomas and showed that gain of 3q can occur in morphologically normal Pap smears of women who developed cervical carcinomas after only a short latency [14], [38]. These findings raised the possibility that *WIG-1* drives 3q gain in cervical cancer. We therefore examined *WIG-1* and its expression in cervical carcinoma cell lines, and assessed Wig-1 expression patient tumor biopsies.

It is clear from our SKY, CGH and Southern blotting analyses of chromosome 3q amplifications in cervical cancer cell lines, that *WIG-1* is not a main target for the frequent gains. *WIG-1* is located telomeric of the commonly amplified 3q23–24 region in Me180 and SiHa cells, and centromeric of the amplified 3q27-ter interval in ME-180 cells. Importantly, the 3q amplifications were not accompanied by *WIG-1* gene amplification as determined by Southern blotting or FISH. Thus, we conclude that *WIG-1* is not the critical gene that drives 3q gain in cervical cancer. Moreover, our analysis of *WIG-1* mRNA and protein levels in the cervical carcinoma cell lines did not reveal any significant correlation between *WIG-1* expression and copy number alterations at the DNA level. We noted, however, that *WIG-1* mRNA expression was higher in the two HPV-negative cervical carcinoma lines (C33-A, HT-3) than in the HPV-positive lines that only showed low or moderate *WIG-1* mRNA levels (Table 2 and Figure S2 in File S1). We did not detect any association between Wig-1 protein expression and HPV infection.

To assess Wig-1 expression in primary cervical tumors, we performed immunohistochemistry on a series of squamous cervical carcinomas (SCC) and adenocarcinomas (ADCA). The prevalence of HPV infection in SCCs is almost 100% [37]. We have previously found HPV-DNA in 70% of ADCA, by using PCR and direct HPV-DNA sequencing [34]. Thus, factors other than the sexually transmitted agent HPV are likely to play a role in the etiology of cervical ADCA. In the current study, we wished to examine the role of Wig-1 in the development of HPV-positive and HPV-negative cervical adenocarcinomas. The higher nuclear Wig-1 expression in the HPV-negative tumors suggests that elevated expression of Wig-1 might play a role in cervical carcinogenesis in the absence of HPV infection. This is consistent with the observation that the cervical carcinoma cell lines carrying high risk HPV had lower Wig-1 mRNA levels compared to the HPV-negative lines.

Interestingly, while all cervical tumors showed positive Wig-1 staining we observed several distinct Wig-1 immunostaining patterns, including moderate nuclear and positive cytoplasmic staining (pattern A) and strong nuclear and negative cytoplasmic staining (pattern B) (Figure 2A–B). We found that pattern B with strong nuclear and negative cytoplasmic Wig-1 staining was associated with poor survival in this group of patients. What could be the biological significance of this association? Our previous results indicate that Wig-1 has a pro-survival function. Wig-1 may stimulate cell proliferation and antagonize cell death through upregulation of putative mRNA targets such as *MYCN*, *c-FOS*, *c-JUN* and *CYCLIN D1*, and downregulation of the pro-apoptotic *FAS* [24], [26], [27]. Thus, it is plausible that high nuclear Wig-1 expression in cervical cancer cells drives cell proliferation through stabilization of pro-growth mRNA targets and destabilization of pro-apoptotic mRNA targets. However, the exact roles of nuclear and cytoplasmic Wig-1 remain to be elucidated.

Since *WIG-1* is a p53 target gene, the question arises as to whether Wig-1 expression levels correlate with p53 status of the tumors and/or presence of HPV, which encodes the E6 protein that targets p53 for degradation. The *TP53* gene is more frequently mutated in HPV-negative than in HPV-positive cervical cancer cell lines and tumors [39]. Among 8 of the cervical cancer cell lines studied here, the only two HPV-negative lines, C-33A and HT-3, both carry *TP53* mutation (Table 2). The *TP53* status of the primary cervical carcinomas in our study has not been determined. Thus, further studies to determine *TP53* status in all tumor samples and to examine the correlation between Wig-1 expression levels and *TP53* status are required. Yet it should be noted that p53 is probably not the only transcription factor that can regulate Wig-1. Indeed, we detected Wig-1 protein in all cervical cell lines irrespective of *TP53* status or genomic gain of the *WIG-1* gene. According to Pscan (http://159.149.109.9/pscan), several other potential transcription factors, for instance *SOX2*, can potentially induce Wig-1 expression [23]. The finding that elevated Wig-1 expression is not correlated with functional p53 indicates that Wig-1 expression is dependent on other mechanisms in addition to direct p53-mediated transcriptional transactivation.

From the clinical standpoint it is very important to identify women at risk of developing cervical cancer by finding early markers that permit rapid and focused preventive action, as well as to elucidate the factors that influence development of cancer in HPV-infected women and HPV-negative women.

It is also important to increase the biological understanding of the factors that influence the development of cervical cancer in women with and without HPV infection, and to understand the molecular mechanisms of cervical carcinogenesis, in order to improve early diagnosis, differential diagnosis, and evaluation of tumor aggressiveness. Molecular markers will help to differentiate between benign disease and malignancy, and thereby prove useful in clinical practice. Our demonstration that Wig-1 expression levels are higher in HPV-negative cervical carcinoma suggests a possible role of Wig-1 in HPV-negative cervical carcinogenesis. Moreover, the finding that moderate nuclear Wig-1 expression levels and positive cytoplasmic Wig-1 staining are associated with better survival indicates that Wig-1 expression levels as assessed by immunohistochemistry could represent a novel prognostic maker for cervical cancer. Our results may contribute to further optimization of current tumor diagnostics and pave the way for the development of new treatment of cervical cancer in the future.

In our study, HPV infection was not detected in all cases, which could either reflect true HPV negativity or represent ‘false negatives’ owing to technical difficulties in HPV genome detection. However, in the current literature, HPV infection has not been reported in the same proportion in adenocarcinomas as in squamous cervical carcinomas [3], [4], [40], [41] and it is very important to find other markers for diagnosis of women at risk for developing cervical adenocarcinomas.

HPV transmission is increasingly common in young women worldwide and several biological factors may help us to develop more effective preventive screening strategies for detection and treatment of women with cervical carcinomas. Should we be able to identify women at risk of developing such cancers based on a single marker, or a combination of different markers, such analyses may become a substitute for, or complement to, cytological screening. Studies indicate that first-generation HPV vaccines might prevent at least two-thirds of cervical cancers. By combining improved detection of precursor lesions of cervical cancer using molecular markers in screening programs with vaccination against HPV, cervical cancer may become the most preventable cancer globally.

## Supporting Information

Table S1 **Clnical and Wig-1 immunohistochemistry data for cervical carcinoma cases in the study.**
(XLS)

File S1 **Figure S1, Western blot analysis of Wig-1 protein expression.** Western lot analysis showing Wig-1 expression levels in eight cervical carcinoma cell lines and one osteosarcoma cell line (Saos2). Beta-actin was used as loading control. * Saos2 was used as a control cell line. **Figure S2,** ***WIG-1*** **mRNA expression in HPV-positive and HPV-negative cervical carcinoma cell lines.** The scatter plot shows relative WIG-1 mRNA expression levelsas assessed by qRT-PCR. HPV-positive lines: Ca Ski, ME-180, MS751, SiHa, SW756 and C-41; HPV-negative lines: C-33A and HT-3. **Figure S3, Specificity of the Wig-1 antibody (Genetex, 1∶2000 dilution).** Western blot analysis showing a single band of expected size at 34 kDa for endogenous Wig-1 in cervical carcinoma tissue. Molecular size markers are shown to the right in kDa. Beta-actin served as a loading control.
(DOC)
