# Supplementary material for: Expression of the p53 Target Wig-1 Is Associated with HPV Status and Patient Survival in Cervical Carcinoma
Source: PLoS One. 2014 Nov 7;9(11):e111125. doi: 10.1371/journal.pone.0111125 (PMC4224373; doi:10.1371/journal.pone.0111125)
Supplement: File S1 — Figure S1, Western blot analysis of Wig-1 protein expression. Western lot analysis showing Wig-1 expression levels in eight cervical carcinoma cell lines and one osteosarcoma cell line (Saos2). Beta-actin was used as loading control. * Saos2 was used as a control cell line. Figure S2, WIG-1 mRNA expression in HPV-positive and HPV-negative cervical carcinoma cell lines. The scatter plot shows relative WIG-1 mRNA expression levelsas assessed by qRT-PCR. HPV-positive lines: Ca Ski, ME-180, MS751, SiHa, SW756 and C-41; HPV-negative lines: C-33A and HT-3. Figure S3, Specificity of the Wig-1 antibody (Genetex, 1∶2000 dilution). Western blot analysis showing a single band of expected size at 34 kDa for endogenous Wig-1 in cervical carcinoma tissue. Molecular size markers are shown to the right in kDa. Beta-actin served as a loading control. (DOC) [file pone.0111125.s002.doc]

**File S1**

**SUPPLEMENTARY MATERIALS**

**HPV typing of established cell lines and tumor samples**

The established cell lines were analysed with a broad-spectrum PCR assay for detection of all HPV types, using degenerate PCR consensus primers MY09 and MY11 located within the conserved L1 region of HPV as previously described . For HPV typing, positive PCR products were sequenced in an ABI 377 DNA Analyzer (Applied Biosystems, Stockholm, Sweden) using the ABI Prism^TM^ BigDye^TM^ Terminator Cycle Sequencing Ready Reaction Big Dye Terminators Kit 3.1 (Applied Biosystems) according to the manufacturer’s protocol. The MY 09 primer was used as the sequencing primer in cycle sequencing.

HPV analysis of the tissues samples have been published for a subset of cases (SCC1-8, and ADCA14-22) by Andersson et al . Identification and typing of HPV were carried out by DNA analyses of extracted DNA obtained from a 10μm-thick section of paraffin blocks. Standard PCRs were carried out, after which GP5+ (5'-TTTGTTACTGTGGTAGATACTAC-3') and GP6+ (3'-CTTATACTAAATGTCAAATAAAAAG-5') primers were applied. The amplified material was then typed by direct PCR DNA sequencing, using consensus primers for sequencing of the two strands, the BigDye Terminator kit (nucleotides, Life technology, Stockholm, Sweden) and the Applied Biosystems 7700 Sequence Detection System. The DNA sequences obtained were compared with known HPV sequence databases using the BLAST algorithm (www.ncbi.nlm.nih.gov./BLAST), with an E-value of < 1× 10^-10^ of a 75 bp alignment accepted as correct typing.

For cases SCC9-13 and ADCA23-38, HPV types were tested using PCR amplification of a fragment in the L1 gene. Samples were tested for the presence of HPV by amplifying 1μl of DNA with the MGP primer system as described . HPV detection and genotyping were conducted on the Luminex Bioplex 200 (Bio-Rad, Stockholm, Sweden) using multiplex bead-based hybridization with Luminex technology as described by Schmitt et al .

**Comparative genomic hybridization (CGH)**

CGH was performed essentially as previously described . Briefly, DNA extracted from the cell lines was labeled with fluorescein-12-dUTP (Perkin Elmer, Upplands Väsby, Sweden) by nick translation, and normal reference DNA was labeled with Spectrum Red (Abbott molecular, Stockholm, Sweden). Cell line and reference DNA were mixed together with unlabeled Cot-1 DNA (Life Technologies, Stockholm, Sweden), denatured and co-hybridized to normal female human metaphase chromosomes (Abbott Molecular). After hybridization at 37°C for 72 hours, the slides were washed and air-dried, after which the chromosomes were counterstained with 4,6-diamidino-2-phenylindole (DAPI; Abbott Molecular) to enable identification. For each hybridization, a control experiment was performed to ensure adequate hybridization conditions, in which normal male genomic DNA (Promega,Stockholm, Sweden) and normal female genomic DNA (Abbott molecular) were co-hybridized to normal metaphases. All cell lines were hybridized twice to control for reproducibility. At least 10 metaphases were captured from each hybridized slide using a Zeiss Axioplan 2 epifluorescence microscope (Carl Zeiss Jena GmbH, Jena, Germany) and documented with the Isis imaging system (Metasystems, Stockholm, Sweden). A green-to-red ratio of less than 0.80 was considered as loss, greater than 1.20 as gain, and above 1.50 as amplification of genetic material. This study focused on the *WIG-1* locus at 3q26.32 and detailed analyses of chromosome 3 were performed and reported.

**Spectral karyotyping (SKY)**

SKY was carried out as previously described according to manufacturer’s protocol (Applied Spectral Imaging, ASI, Migdal Haemek, Israel). Slides with metaphase cells from the lines were prepared according to standard protocols. Briefly, after culturing to 70% confluence, the cells were treated with Colcemid (Sigma, Stockholm, Sweden), harvested, fixed and dropped on clean slides. Image acquisitions were performed using the SD200 Spectracube system (ASI) mounted on a Zeiss Axioskop epifluorescence microscope with a custom-designed optical filter (SKY-1, Chroma Technology, Brattleboro, VT, USA). SKY View 1.5 software (ASI) was used for spectral classification of the raw spectral images. After SKY karyotyping, all chromosomes containing material from chromosome 3 were analyzed in detail. Clonality criteria and karyotype descriptions were based on the recommendations of the International System for Human Cytogenetic Nomenclature .

**Southern blot analysis**

Southern analysis was performed on 7 of the cell lines using a probe corresponding to the entire open reading frame of human *WIG-1* and a control probe from an anonymous region in 3p23. The control probe was generated by PCR amplification from human genomic DNA using the primers 3pF (5'- GTGCTATGCCTAACTCATA TTA-3') and 3pR (5'- GCCACAACAACTATTTTCCAA-3'). Southern analysis was carried out according to standard protocol. Genomic DNA was extracted from cells using standard protocol with Proteinase K digestion, phenol/chloroform extraction and ethanol precipitation. Ten μg of each DNA sample were cleaved with the restriction endonuclease *Eco*RI, separated by electrophoresis in 0.8% agarose gel and transferred to nylon filter (GE Healthcare, Uppsala, Sweden). A 50 ng aliquot of each probe was labeled with ^32^P-dCTP using Ready-To-Go DNA Labelling Beads (GE Healthcare, Uppsala, Sweden) and hybridized to the prehybridized filter. The ratio between the signals obtained using the *WIG-1* probe and the 3p23 control probe was determined for each sample through visual inspection by three independent observers. DNA from peripheral blood leukocytes from a healthy donor was included as a normal reference.

**LightCycler quantification of *WIG-1* expression (qRT-PCR)**

Total RNA was extracted from freshly harvested cells with Trizol reagent (Life Technologies) and followed by DNase I treatment. Extracted total RNA was quantified and purity was assessed by visualization in 1% agarose gel. The High-Capacity cDNA Archive Kit (ABI, CA, USA) was used for cDNA synthesis by reverse transcription (25 °C, 10 minutes; 42 °C, 120 minutes). The reaction was carried out using 1.5 μg of total RNA with 10 × RT buffer, 25 × dNTP mixture, 10 × random primers, MultiScribe reverse transcriptase and RNAse-free molecular grade water in a total volume of 100 μL, as per protocol. The assay for expression was set up using the DNA Engine Opticon Real-Time PCR detection system labeled with SYBR@Green I dye (Bio-Rad, Stockholm, Sweden). *HPRT1* (Life Technologies, Stockholm, Sweden) was used as a reference gene for the assay. Gene-specific primers were designed for *WIG-1* based on mRNA sequence and purchased (Sigma-Aldrich, Stockholm, Sweden). Left primer sequence: CTCTCGGCAGAGAATTCCAC (position 681-700); Right primer: CCTGTACCGCTGTTCAGACA (position 1099-1080), (positions in cds, according to cds database 3224.1). Certified LUX^TM^ Primer Sets of hHPRT1 (Gene Acc.No.NM\_000194 Reverse labeled FAM cat.no. 105H-01, JOE cat.no. 105H-02, Invitrogen, Stockhom, Sweden) were used to amplify hHPRT1. PCR reactions were carried out in 50μl MJ white tube sets in replicates using 96-well plate set-ups with no-template controls and standards. The LightCycler PCR and detection system was used for amplification and online quantification. The iQ SuperMix (Bio-Rad, Stockholm, Sweden) was used for amplicon detection. A total volume of 20 μL of PCR mixture was prepared using 10μL 2 × supermix (Taq polymerase, light cycler hybridization buffer, SYBR@Green I dye with dUTP, and 3 mM magnesium chloride), 5 μL of primer mix (final conc. 250 nM) and 5 μL of suitably diluted cDNA product. After initial Taq activation at 95 °C for 10 minutes, LightCycler PCR was performed under the following four conditions: 95 °C for 15 seconds, 60°C for 5 seconds, 72 °C for 10 seconds, and 83 °C for 5 seconds. Quantification was performed by online monitoring to identify the exact time point at which the logarithmic linear phase could be distinguished from the background (threshold). Serially diluted cDNA samples were used as standards. cDNA template concentration was calculated by comparing the cycle number of the logarithmic linear phase of this sample with the cycle numbers of the external standards. *WIG-1* gene quantification was expressed in arbitrary units in relation to the reference gene. RNA transcripts from normal human fibroblasts were used as controls.

Reference

Andersson S, Rylander E, Larson B, Sigurdardottir S, Backlund I, Sallstrom J, Wilander E (2003) Types of human papillomavirus revealed in cervical adenocarcinomas after DNA sequencing. Oncol Rep 10(1): 175-9

Farnebo F, Kytola S, Teh BT, Dwight T, Wong FK, Hoog A, Elvius M, Wassif WS, Thompson NW, Farnebo LO, Sandelin K, Larsson C (1999) Alternative genetic pathways in parathyroid tumorigenesis. The Journal of clinical endocrinology and metabolism 84(10): 3775-80

Herrington CS, McGee JOD (1992) Diagnostic Molecular Pathology: Cell and tissue genotyping. Vol. 2: Oxford University Press, USA

Kytola S, Rummukainen J, Nordgren A, Karhu R, Farnebo F, Isola J, Larsson C (2000) Chromosomal alterations in 15 breast cancer cell lines by comparative genomic hybridization and spectral karyotyping. Genes, chromosomes & cancer 28(3): 308-17

Manos M, Lee K, Greer C, Waldman J, Kiviat N, Holmes K, Wheeler C (1990) Looking for human papillomavirus type 16 by PCR. Lancet 335(8691): 734

Mitelman F (1995) ISCN 1995: an international system for human cytogenetic nomenclature (1995): recommendations of the International Standing Committee on Human Cytogenetic Nomenclature, Memphis, Tennessee, USA, October 9-13, 1994: Karger Basel:

Schmitt M, Bravo I, Snijders PJ, Gissmann L, Pawlita M, Waterboer T (2006) Bead-based multiplex genotyping of human papillomaviruses. J Clin Microbiol 44(2): 504-512

Soderlund-Strand A, Carlson J, Dillner J (2009) Modified general primer PCR system for sensitive detection of multiple types of oncogenic human papillomavirus. J Clin Microbiol 47(3): 541-6

**Figure S1. Western blot analysis of Wig-1 protein expression.** Western lot analysis showing Wig-1 expression levels in eight cervical carcinoma cell lines and one osteosarcoma cell line (Saos2). Beta-actin was used as loading control.

* Saos2 was used as a control cell line.

**Figure S2. *WIG-1* mRNA expression in HPV-positive and HPV-negative cervical carcinoma cell lines.** The scatter plot shows relative WIG-1 mRNA expression levelsas assessed by qRT-PCR. HPV-positive lines: Ca Ski, ME-180, MS751, SiHa, SW756 and C-41; HPV-negative lines: C-33A and HT-3.


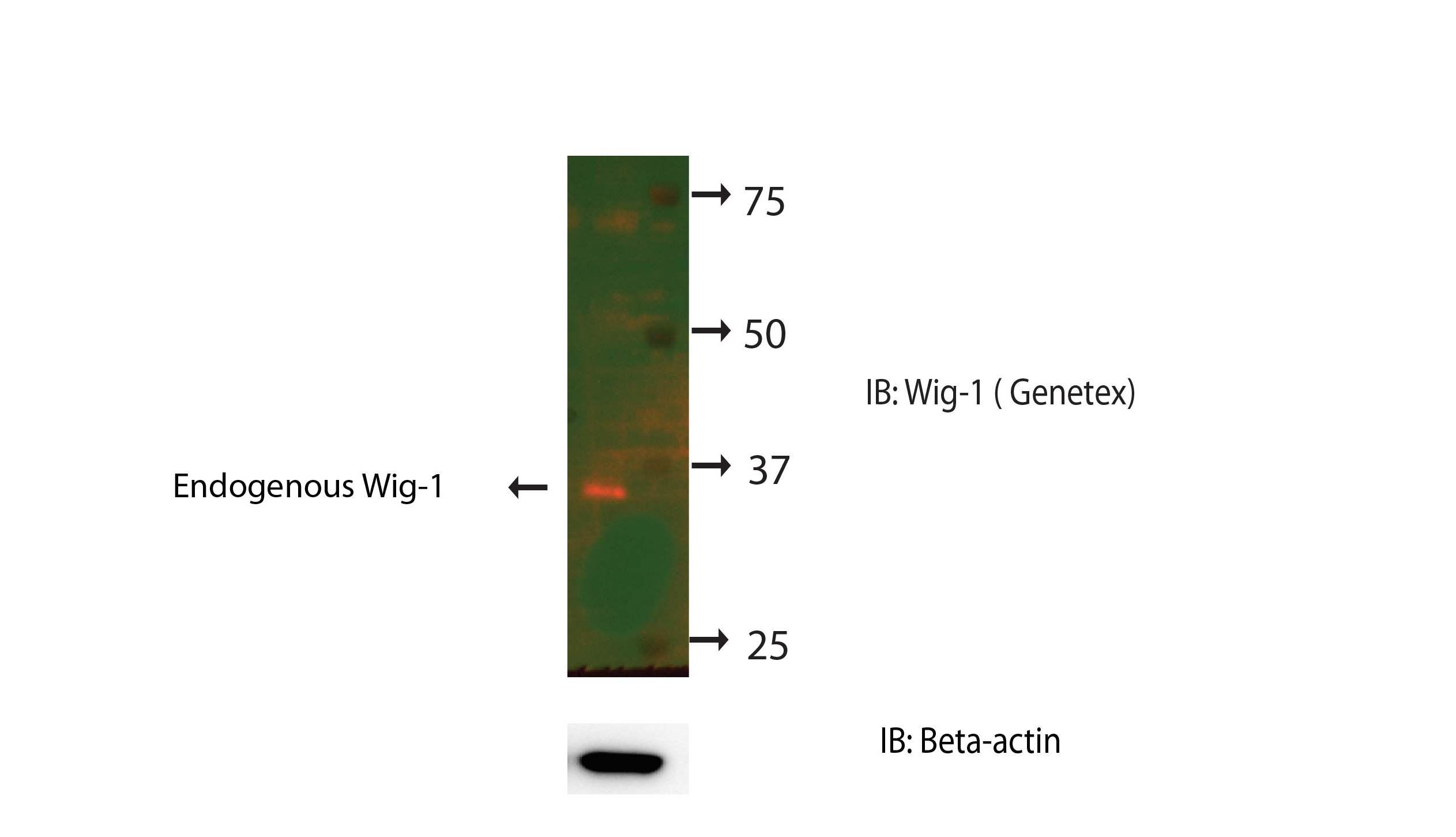


**Figure S3. Specificity of the Wig-1 antibody (Genetex, 1:2000 dilution).** Western blot analysis showing a single band of expected size at 34 kDa for endogenous Wig-1 in cervical carcinoma tissue. Molecular size markers are shown to the right in kDa. Beta-actin served as a loading control.
